# Predictive value of preoperative and postoperative controlling nutritional status scores and their changes in older patients with large vessel occlusion stroke after endovascular thrombectomy

**DOI:** 10.3389/fnut.2026.1838003

**Published:** 2026-08-05

**Authors:** Shiyuan Tian, Xuefang Ma, Xiaoyang Liu, Furong Shang, Jing Yang

**Affiliations:** 1 Department of Neurology, Xiangyang Central Hospital, Affiliated Hospital of Hubei University of Arts and Science, Xiangyang, Hubei, China; 2 Xiangyang Central Hospital, Affiliated Hospital of Hubei University of Arts and Science, Xiangyang, Hubei, China; 3 Department of Neurology, China-Japan Union Hospital of Jilin University, Changchun, Jinlin, China

**Keywords:** controlling nutritional status score, endovascular thrombectomy, large vessel occlusion, nomogram, predictors

## Abstract

**Background:**

Over the past decade, endovascular thrombectomy (EVT) for acute large vessel occlusion (LVO) has advanced significantly. However, older patients who present with large-vessel occlusion and are treated with EVT tend to have poorer outcomes compared to younger individuals. This study aims to investigate whether preoperative and postoperative controlling nutritional status (CONUT) scores can predict clinical outcomes and their dynamic changes in older patients with large vessel occlusion stroke following EVT.

**Methods:**

A total of 441 patients with LVO who underwent EVT were enrolled from two stroke units. These patients were divided into a prospective training cohort and a prospective validation cohort. Based on the 90-day modified Rankin Scale (mRS) score, patients were categorized into either a favorable outcome group or an unfavorable outcome group. Univariate analysis and logistic regression were used to screen candidate predictors. A nomogram was developed to predict the risk of unfavorable outcomes. The predictive performance of the model was evaluated using receiver operating characteristic (ROC) curve analysis. Model calibration was assessed with the Hosmer–Lemeshow (H-L) test and calibration curves. Decision curve analysis (DCA) was applied to evaluate the clinical utility of the model.

**Results:**

Multivariable logistic regression analysis revealed that admission NIHSS (OR = 1.096; 95% CI: 1.031–1.166; *p* = 0.005), ASPECTS on NCCT (OR = 0.598; 95% CI: 0.426–0.839; *p* = 0.001), LKW-to-puncture time (OR = 1.017; 95% CI: 1.012–1.022; *p* < 0.001) and CONUT (OR = 1.498; 95% CI: 1.265–1.773; *p* < 0.001) were independent predictors of unfavorable 90-day outcomes after EVT in older patients with LVO. The nomogram constructed using these four factors achieved an area under the curve (AUC) of 0.839. The calibration curve closely approximated the ideal diagonal line. Furthermore, DCA demonstrated a significantly higher net benefit of the model across a range of threshold probabilities. External validation confirmed the reliability of the prediction nomogram.

**Conclusion:**

The CONUT score-based nomogram model demonstrates comparable predictive performance for predicting adverse outcomes at 90 days in older patients with LVO undergoing EVT, whether applied preoperatively or postoperatively. This tool provides clinicians with an accurate and effective method for early prediction and timely treatment planning in this specific patient population.

## 1 Introduction

Large Vessel Occlusion (LVO) is a major cause of disability and mortality in ischemic stroke and represents a substantial global disease burden. With the global population aging at an unprecedented rate, the incidence and mortality of stroke are projected to rise accordingly (1). Consequently, the management of stroke in older patients presents substantial challenges to healthcare systems and society.

Early recanalization of occluded vessels to salvage the ischemic penumbra remains the cornerstone of acute ischemic stroke (AIS) management. Endovascular thrombectomy (EVT) has emerged as a standard and effective treatment for AIS resulting from LVO (2). However, older patients may experience poorer clinical outcomes following EVT compared to their younger counterparts. This is attributable not only to the decline in biological function associated with aging but also to technical challenges posed by severe arteriosclerosis and vascular tortuosity. While such factors are often non-modifiable, other elements—such as the nutritional status of older patients—remain amenable to intervention.

Malnutrition represents a common yet often under recognized concern in the older population. Among patients hospitalized with acute ischemic stroke (AIS), the prevalence of malnutrition present on admission has been reported to reach approximately one-third (ranging from 33 to 34.3%) (3, 4). Growing evidence further indicates that malnutrition at admission is independently associated with worse clinical outcomes in AIS, including higher mortality and poorer neurological function at 90 days after onset (5, 6). Consequently, systematic early nutritional screening is imperative in this patient group to facilitate timely and tailored nutritional interventions.

In recent years, multiple studies have demonstrated that impaired preoperative nutritional status, as assessed by the Controlling Nutritional Status (CONUT) score, serves as an independent risk factor for both short and long-term outcomes in patients undergoing endovascular coronary or peripheral arterial interventions (7, 8). The CONUT score is calculated from the serum albumin level, peripheral lymphocyte count, and the total cholesterol (TC) concentration. This score can be calculated using only routine blood tests and objectively reflects a patient’s nutritional status.

It remains unclear whether preoperative and postoperative CONUT scores can predict clinical outcomes and their dynamic changes in older patients with large vessel occlusion stroke following EVT. This study aims to develop a prognostic nomogram based on controlling nutritional status score to predict poor outcomes after EVT in older patients with LVO stroke, and to validate its clinical applicability, while also examining the impact of dynamic changes in CONUT on the predictive performance of the nomogram model. The research will contribute to enhancing the precision and overall effectiveness of EVT in treating older patients with ischemic stroke, thereby supporting more personalized treatment strategies.

## 2 Materials and methods

### 2.1 Study design and population

Our study enrolled 441 patients with LVO who underwent EVT between January 2024 and July 2025 at Xiangyang Central Hospital and China-Japan Union Hospital of Jilin University. The cohort was divided into two distinct groups: a prospective training cohort comprising 316 patients consecutively treated at Xiangyang Central Hospital from January 2024 to December 2024, and a prospective external validation cohort consisting of 125 patients treated at China-Japan Union Hospital of Jilin University from January 2025 to July 2025. The study was conducted in compliance with the ethical standards described in the Declaration of Helsinki. Ethical approval was granted by the Ethics Committee of Xiangyang Central Hospital (Approval No. 2024–054) and the Ethics Committee of China-Japan Union Hospital of Jilin University (Approval No. 2025011505). All participants and their legal guardians provided informed consent. The inclusion criteria were as follows: (1) age ≥65 years; (2) confirmation of LVO via non-contrast computed tomography (NCCT) and computed tomography angiography (CTA); (3) time from last known well (LKW) to arterial puncture <24 h; (4) baseline mRS score ≤2 and admission National Institutes of Health Stroke Scale (NIHSS) score ≥6. Exclusion criteria were: (1) incomplete medical records or missing data; (2) lost to follow-up at 3 months.

### 2.2 Data collection

The demographic and clinical variables encompassed age, sex, smoking status, alcohol consumption, hypertension, diabetes mellitus, atrial fibrillation, hyperlipidemia, history of prior stroke, medication history, baseline mRS, admission NIHSS score, admission Alberta Stroke Program Early CT Score (ASPECTS), time from last known well (LKW) to puncture, time from puncture to recanalization, perfusion imaging parameters, modified Thrombolysis in Cerebral Infarction (mTICI) grade, Trial of Org 10,172 in Acute Stroke Treatment (TOAST) classification, number of thrombectomy attempts, and 90-day mRS.

### 2.3 Nutritional status assessment

Venous blood was drawn for laboratory analysis before anesthesia induction and within 24 h after EVT. The CONUT score was estimated using serum albumin concentration, peripheral lymphocyte count, and the total cholesterol concentration (Supplementary Table S1). The CONUT score range is 0–12. Based on the score, the nutrition status can be classified into four levels: absent (0–1), mild (2-4), moderate (5-8), and severe (9-12) (4).

### 2.4 Therapeutic strategy

Patients who met the eligibility criteria for intravenous thrombolysis were administered intravenous alteplase at a dose of 0.9 mg/kg prior to EVT. All procedures were performed under general anesthesia. The initial EVT strategy employed a combined approach using a stent retriever with intermediate catheter aspiration, known as the SWIM technique. Postoperative digital subtraction angiography (DSA) was performed to assess reperfusion. If blood flow was not adequately maintained, rescue interventions such as balloon angioplasty or stent placement would be considered.

### 2.5 Outcome definitions

The primary outcome measure was functional independence at 90 days, defined as a mRS score of 0–2. Successful reperfusion was characterized by a mTICI score of ≥2b.

### 2.6 Development and assessment of the nomogram

A multivariate logistic regression analysis was performed to develop a nomogram for predicting unfavorable outcomes. Independent predictors (*p* < 0.05), identified through the multivariate regression, were incorporated into the nomogram using the dataset for predicting the occurrence of unfavorable outcomes. A linear regression model was used to analyse the collinearity between the variables. Collinearity severity was quantified using the variance inflation factor (VIF) and the tolerance value, with thresholds set at VIF < 10 and tolerance >0.1, indicating acceptable levels of collinearity. Predictor variable lines were extended upward to assign points for each factor. The total points were then calculated and plotted on the “Total Points” axis. A line was drawn downward from this total to the risk scale to estimate the probability of unfavorable outcomes. The resulting visual prediction model was subsequently subjected to external validation. The Hosmer–Lemeshow test was employed to evaluate model fit. Predictive accuracy and consistency were assessed using the receiver operating characteristic (ROC) curve, the area under the ROC curve (AUC), and calibration curves. Decision curve analysis (DCA) was conducted to quantify the net benefit of the model across different threshold probabilities. Both discriminative ability and calibration were validated via bootstrapping with 1,000 resamples. The dynamic changes in CONUT were defined as ∆CONUT, calculated as the postoperative CONUT score minus the preoperative CONUT score. The preoperative nomogram model was designated as model 1, the postoperative CONUT score combined with other selected predictive factors as model 2, and ∆CONUT combined with other selected predictive factors as model 3. The predictive performance of the three models was compared using ROC curves. Comparison of the ROC curves was based on the method of DeLong et al. (9).

### 2.7 Statistical analysis

Data were analysed with SPSS statistics (version 25.0) and R (version 4.1.2). Statistical significance was set at *p* < 0.05. Continuous variables were represented as mean ± standard deviation or median (interquartile range) and were compared using either Student’s *t*-test or the Mann–Whitney *U* test, contingent upon the normality of the data distribution. We employed multiple imputation using chained equations (MICE) to handle missing continuous data. Categorical variables were depicted as frequencies (percentages) and were analyzed using the chi-square test or Fisher’s exact test.

## 3 Results

### 3.1 Patients characteristics

**Figure 1:**
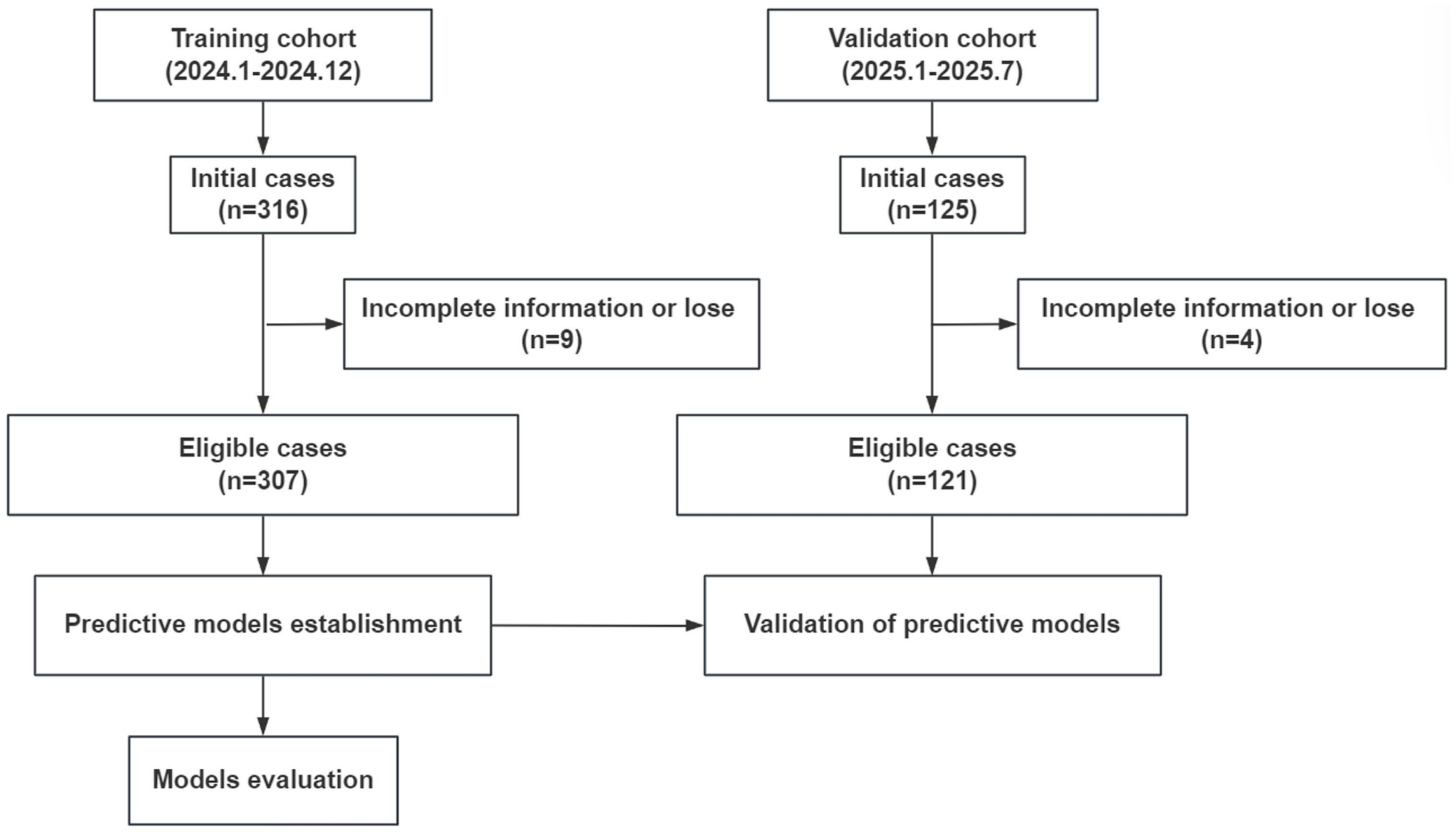
Flow chart for patient selection.

In the training cohort, 9 patients (2.8%) were lost to follow-up, compared with 4 (3.2%) in the external validation cohort. Among the remaining 428 patients, 307 were included in the training cohort and 121 in the external validation cohort (Figure 1). The balanced demographic and clinical characteristics of 307 patients in the training cohort and 121 patients in the validation cohort are shown in Supplementary Table S2. Based on the 90-day mRS scores, 115 patients (115/307, 37.5%) admitted to Xiangyang Central Hospital were classified into the favorable outcome group (mRS 0–2), while 192 patients (192/307, 62.5%) were assigned to the unfavorable outcome group (mRS > 2). The unfavorable outcome group exhibited elevated admission NIHSS (0.004), reduced ASPECTS on NCCT (*p*<0.001), higher proportion of mTICI < 2b (*p* = 0.03), prolonged LKW-to-puncture time (*p*<0.001), higher rates of malnutrition (*p*<0.001) (Table 1).

**Table 1:** Comparison of baseline data in patients with favorable versus unfavorable outcomes at 90 days.

Variables	Total (*n* = 307)	Favorable outcome (*n* = 115)	Unfavorable outcome (*n* = 192)	*p*-value
Age, years, M (Q₁, Q₃)	71.50 (68.00, 74.00)	71.00 (69.00, 74.00)	72.00 (68.00, 74.00)	0.690
Gender, *n* (%)				0.341
Female	97 (31.60)	33 (28.70)	64 (33.33)	
Male	210 (68.40)	82 (71.30)	128 (66.67)	
Hypertension, *n* (%)				0.055
No	89 (28.99)	41 (35.65)	48 (25.00)	
Yes	218 (71.01)	74 (64.35)	144 (75.00)	
Diabetes mellitus, *n* (%)				0.132
No	240 (78.18)	95 (82.61)	145 (75.52)	
Yes	67 (21.82)	20 (17.39)	47 (24.48)	
Atrial fibrillation, *n* (%)				0.242
No	199 (64.82)	79 (68.70)	120 (62.50)	
Yes	108 (35.18)	36 (31.30)	72 (37.50)	
Hyperlipidemia, *n* (%)				0.280
No	247 (80.46)	96 (83.48)	151 (78.65)	
Yes	60 (19.54)	19 (16.52)	41 (21.35)	
Previous ischaemic stroke, n (%)				0.392
No	238 (77.52)	92 (80.00)	146 (76.04)	
Yes	69 (22.48)	23 (20.00)	46 (23.96)	
Smoking, *n* (%)				0.839
No	190 (61.89)	70 (60.87)	120 (62.50)	
Yes	117 (38.11)	45 (39.13)	72 (37.50)	
Alcohol abuse, *n* (%)				0.647
No	250 (81.43)	95 (82.61)	155 (80.73)	
Yes	57 (18.57)	20 (17.39)	37 (19.27)	
Medication history, *n* (%)				
Pre-statin use				0.637
No	242 (78.83)	93 (80.87)	149 (77.60)	
Yes	65 (21.17)	22 (29.13)	43 (22.40)	
Pre-antiplatelet use				0.168
No	236 (76.87)	94 (81.74)	142 (73.96)	
Yes	71 (23.13)	21 (18.26)	50 (26.04)	
Pre-anticoagulant use				0.921
No	271 (88.27)	102 (88.70)	169 (88.02)	
Yes	36 (11.73)	13 (11.30)	23 (11.98)	
Admission NIHSS, M (Q₁, Q₃)	15.00 (12.00, 19.00)	14.00 (12.00, 17.00)	16.00 (12.00, 20.00)	**0.004**
ASPECTS on NCCT, M (Q₁, Q₃)	10.00 (9.00, 10.00)	10.00 (10.00, 10.00)	10.00 (8.00, 10.00)	**<0.001**
Intravenous thrombolysis, *n* (%)				0.164
No	185 (60.26)	64 (55.65)	121 (63.02)	
Yes	122 (39.74)	51 (44.35)	71 (36.98)	
TOAST classification, *n* (%)				0.388
Large-artery atherosclerosis	159 (51.79)	65 (56.52)	94 (48.96)	
Cardioembolic	115 (37.46)	39 (33.91)	76 (39.58)	
Other etiology	33 (10.75)	11 (9.57)	22 (11.46)	
Vascular occlusion site, *n* (%)				0.960
Internal carotid artery	106 (34.53)	40 (34.78)	66 (34.38)	
Middle cerebral artery	160 (52.12)	58 (50.43)	102 (53.13)	
Vertebral artery	12 (3.91)	5 (4.35)	7 (3.65)	
Basilar artery	29 (9.44)	12 (10.44)	17 (8.84)	
Number of thrombectomy attempts, M (Q₁, Q₃)	1.00 (1.00, 2.00)	1.00 (1.00, 2.00)	1.00 (1.00, 2.00)	0.065
Salvage therapy, *n* (%)				0.383
No	192 (62.54)	68 (59.13)	124 (64.58)	
Yes	115 (37.46)	47 (40.87)	68 (35.42)	
mTICI 2b-3 reperfusion, n (%)				**0.030**
No	20 (6.51)	3 (2.61)	17 (8.85)	
Yes	287 (93.49)	112 (97.39)	175 (91.15)	
LKW-to-puncture time, min, M (Q₁, Q₃)	281.00 (227.00, 329.50)	243.00 (198.00, 275.00)	315.00 (266.00, 362.00)	**<0.001**
Puncture-to-Recanalization time, min, M (Q₁, Q₃)	100.50 (68.00, 144.75)	88.00 (65.00, 148.00)	102.00 (68.00, 143.00)	0.774
CONUT, M (Q₁, Q₃)	2.00 (1.00, 4.00)	1.00 (1.00, 3.00)	3.00 (2.00, 5.00)	**<0.001**
CONUT scoring system, *n* (%)				**<0.001**
Absent	101 (32.90)	59 (51.30)	42 (21.88)	
Mild	132 (43.00)	47 (40.87)	85 (44.27)	
Moderate	71 (23.13)	9 (7.83)	62 (32.29)	
Severe	3 (0.97)	0 (0.00)	3 (1.56)	

CONUT, Controlling nutritional status score; NIHSS, National institute of health stroke scale; ASPECTS. Alberta stroke program early computed score; NCCT, non-contrast computed tomography; TOAST, Trial of org 10,172 in acute stroke treatment; mTICI, modified thrombolysis in cerebral infarction; LKW, last known well. Bold values are categorical data, expressed as percentages.

### 3.2 Screening for predictive factors

In the univariate logistic regression analysis, variables with a *p*-value < 0.2 were selected for inclusion in the multivariate logistic regression analysis. Prior to multivariate regression analysis, collinearity diagnostics indicated that all covariates remained within acceptable multicollinearity thresholds: hypertension (VIF: 1.035, Tolerance: 0.966), admission NIHSS (VIF: 1.088, Tolerance: 0.919), ASPECTS on NCCT (VIF: 1.097, Tolerance: 0.911), intravenous thrombolysis (VIF: 1.131, Tolerance: 0.884), LKW-to-puncture time (VIF: 1.075, Tolerance: 0.930), number of Thrombectomy (VIF: 1.066, Tolerance: 0.938), mTICI 2b-3 reperfusion (VIF: 1.156, Tolerance: 0.865) and CONUT (VIF: 1.164, Tolerance: 0.859). These values suggest that multicollinearity is unlikely to affect the reliability of the regression estimates. In multivariable logistic regression analysis, admission NIHSS (OR = 1.096; 95% CI: 1.031–1.166; *p* = 0.005), ASPECTS on NCCT (OR = 0.598; 95% CI: 0.426–0.839; *p* = 0.001), LKW-to-puncture time (OR = 1.017; 95% CI: 1.012–1.022; *p* < 0.001) and CONUT (OR = 1.498; 95% CI: 1.265–1.773; *p* < 0.001) were independent predictors for a 90-day unfavorable outcome (Table 2).

**Table 2:** Logistic regression analysis of factors associated with unfavorable outcomes.

Variables	Univariate		Multivariate	
	OR (95% CI)	*P*-value	OR (95% CI)	*P*-value
Hypertension	1.630 (1.288–2.289)	0.056		
Admission NIHSS	1.074 (1.022–1.127)	**0.003**	1.096 (1.031–1.166)	**0.005**
ASPECTS on NCCT	0.613 (0.482–0.780)	**<0.001**	0.598 (0.426–0.839)	**0.001**
Intravenous thrombolysis	0.619 (0.451–0.946)	**0.165**		
LKW-to-puncture time	1.018 (1.013–1.023)	**<0.001**	1.017 (1.012–1.022)	**<0.001**
Number of Thrombectomy	1.202 (1.071–1.487)	**0.091**		
mTICI 2b-3 reperfusion	0.330 (0.122–0.893)	**0.029**		
CONUT	1.534 (1.334–1.763)	**<0.001**	1.498 (1.265–1.773)	**<0.001**

CONUT, Controlling nutritional status score; NIHSS, National institute of health stroke scale; ASPECTS. Alberta stroke program early computed score; NCCT, non-contrast computed tomography; mTICI, modified thrombolysis in cerebral infarction; LKW, last known well. Bold values are categorical data, expressed as percentages.

### 3.3 Development and evaluation of the nomogram model in the training cohort

**Figure 2:**
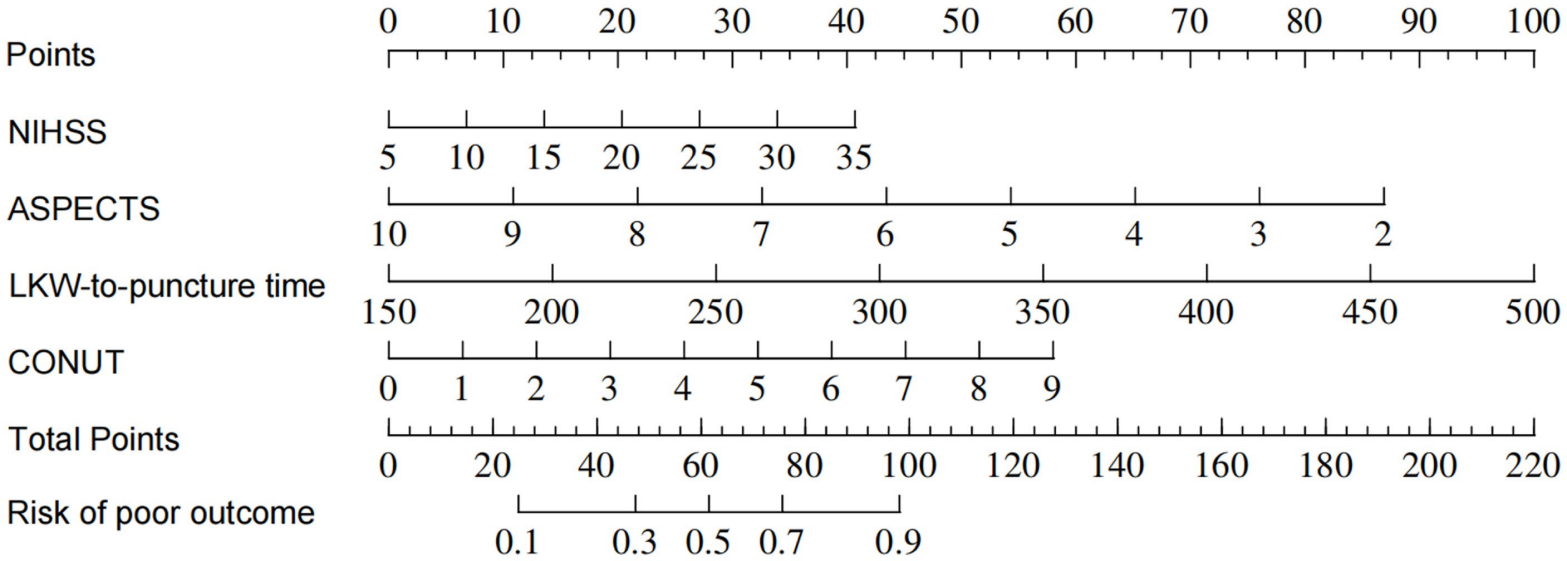
Nomogram for predicting the efficacy of unfavorable outcomes in the training cohort. NIHSS, national institute of health stroke scale; ASPECTS, alberta stroke program early computed score; LKW, last known well; CONUT, controlling nutritional status score.

**Figure 3:**
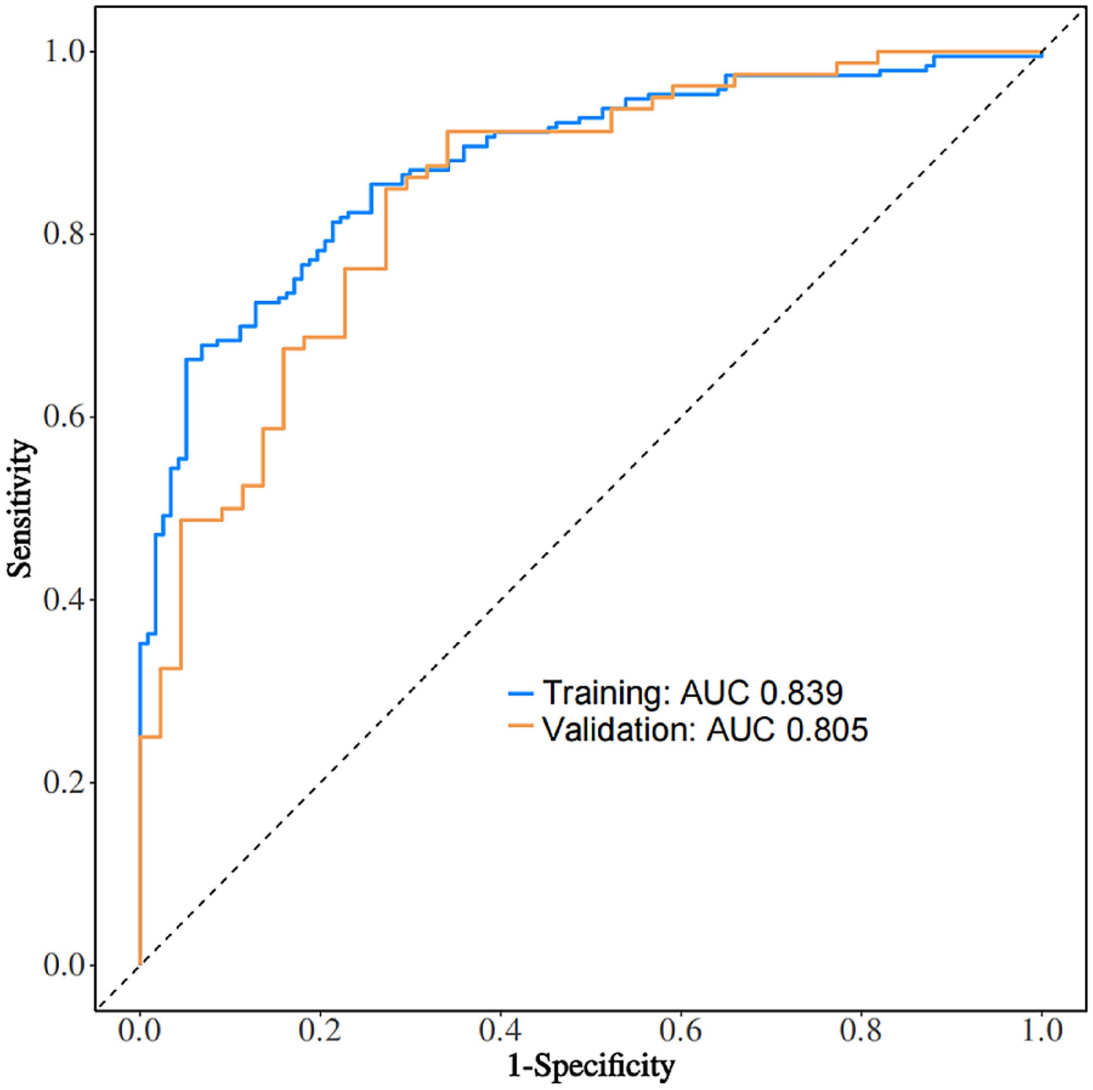
ROC curves. ROC, receiver operating characteristic; AUC, area under the ROC curve.

**Figure 4:**
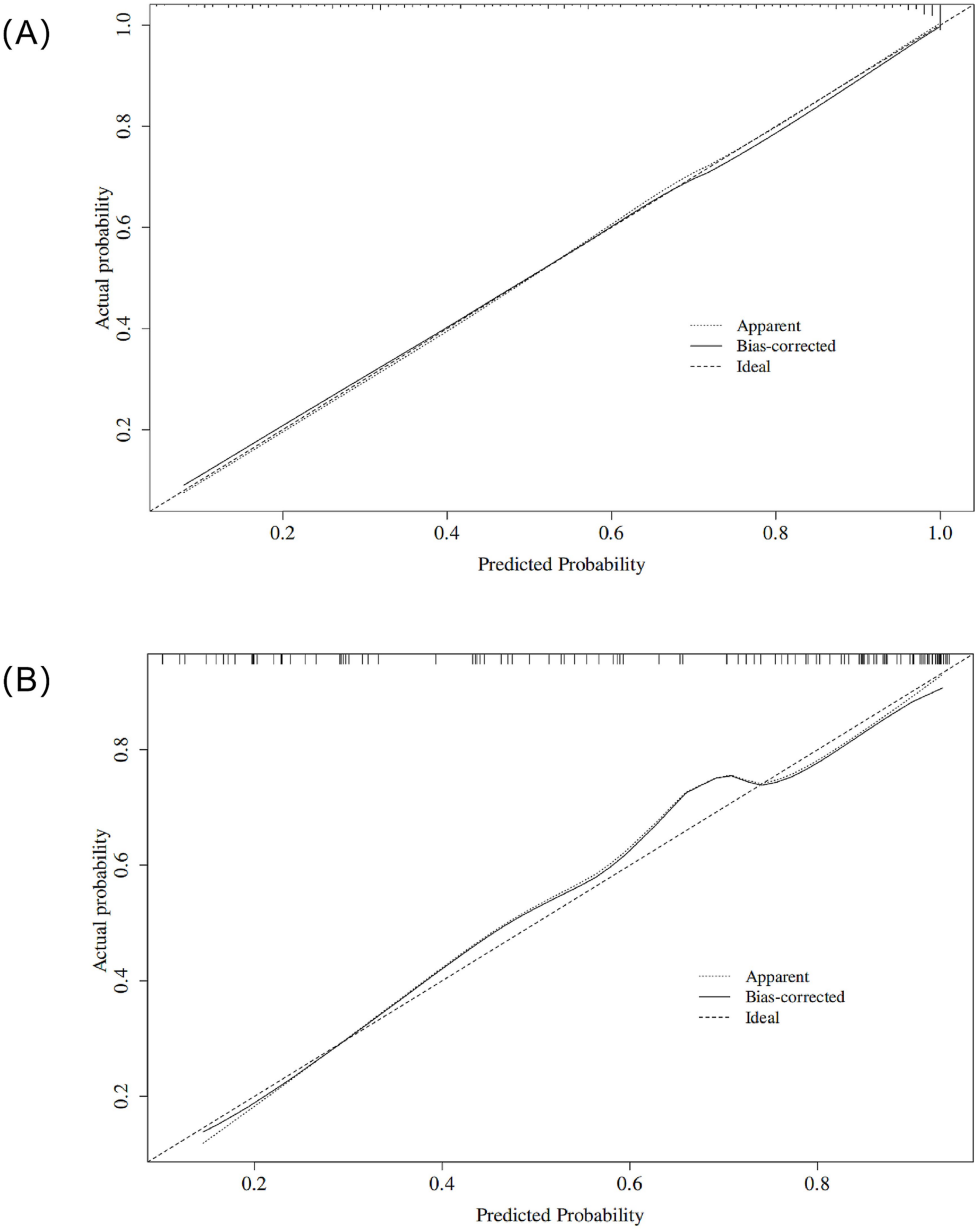
Calibration curve for predicting unfavorable outcomes. **(A)** Training cohort. **(B)** Validation cohort.

**Figure 5:**
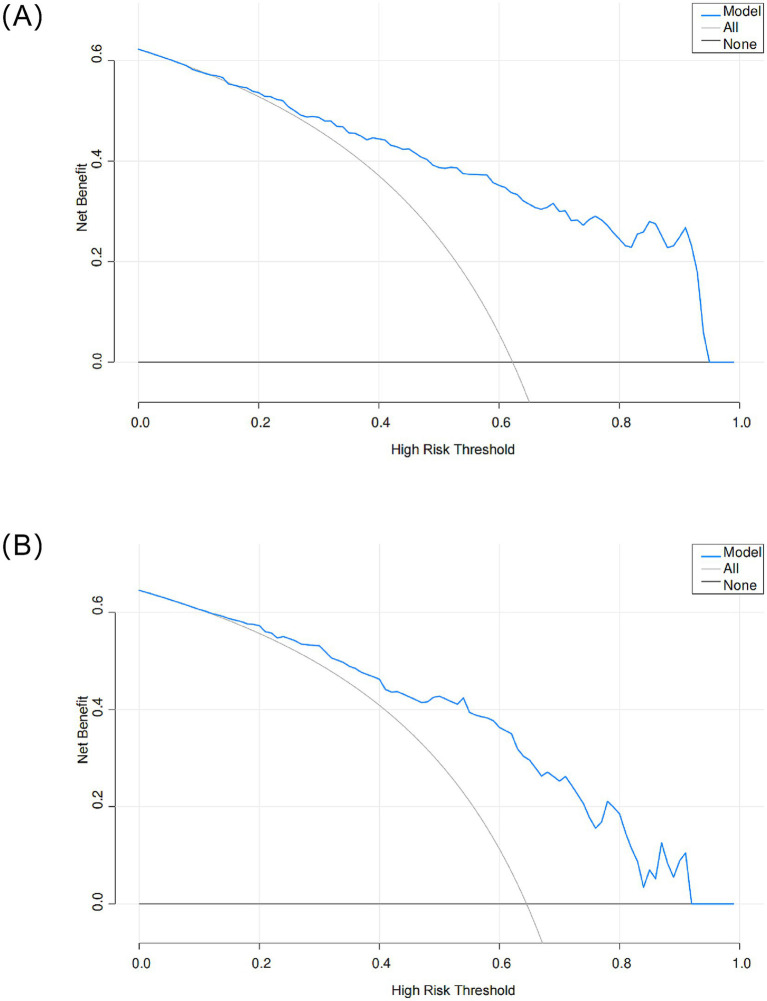
Decision curve analysis in prediction of unfavorable outcomes. **(A)** Training cohort. **(B)** Validation cohort.

The predictive model containing admission NIHSS, ASPECTS on NCCT, LKW-to-puncture time and CONUT was developed and presented as the nomogram (Figure 2). Therefore, if a patient with an admission NIHSS score of 15, an ASPECTS of 10, an LKW-to-treatment time of 300 min, and a CONUT score of 3, the corresponding points on the nomogram are 13.6, 0, 42.97, and 19.46, respectively, resulting in a total of 76.03 points. The corresponding risk of unfavorable outcome at this total point value is approximately 0.7, indicating an estimated unfavorable outcome of 70% for this case. A higher total point value indicates a greater risk of unfavorable outcome. The area under the curve (AUC) of the receiver operating characteristic (ROC) curve was 0.839 in the training cohort (Figure 3), with a cutoff value of 4 for the preoperative CONUT score in predicting unfavorable outcomes. The model demonstrated a sensitivity of 66%, a specificity of 95%, and an accuracy of 77%. A calibration curve for the nomogram in training cohort showed a strong alignment between the calibration and standard curves (Figure 4A). The Hosmer-Lemeshow test demonstrated a strong consistency between the prediction model and the observed outcomes (*p* = 0.963). Furthermore, the decision curve analysis (DCA) showed significantly better net benefit in the predictive model (Figure 5A).

### 3.4 Validation of the nomogram model in the external validation cohort

In addition, an external validation cohort comprising 121 patients from China-Japan Union Hospital of Jilin University was used to evaluate the nomogram. The AUC was 0.805 (Figure 3), reflecting a good accuracy of the nomogram. Meanwhile, the model had good consistency, and the calibration curve of the validation cohort was also close to the ideal diagonal line (Hosmer-Lemeshow *p* = 0.523) (Figure 4B). Moreover, the DCA showed a significant net benefit in the validation cohort as well (Figure 5B). Overall, these results support the potential of the nomogram as a valuable tool for clinical decision-making.

### 3.5 The influence of dynamic changes in the CONUT score on predictive efficacy

**Figure 6:**
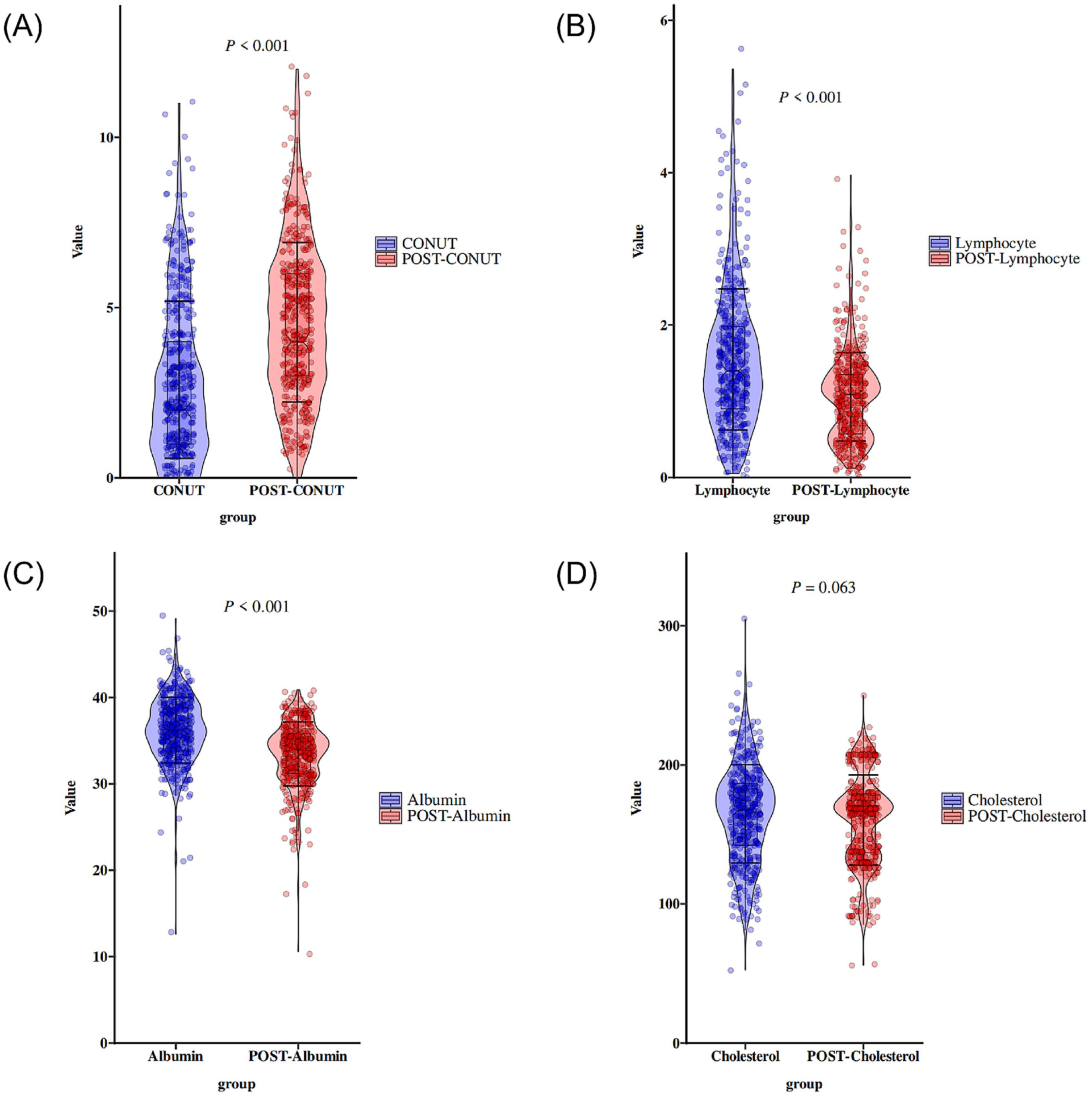
Comparison of preoperative and postoperative CONUT score, lymphocyte count, albumin, and total cholesterol. CONUT, Controlling Nutritional Status score. Violin plot **(A)** CONUT score; **(B)** lymphocyte count; **(C)** albumin; **(D)** total cholesterol.

**Figure 7:**
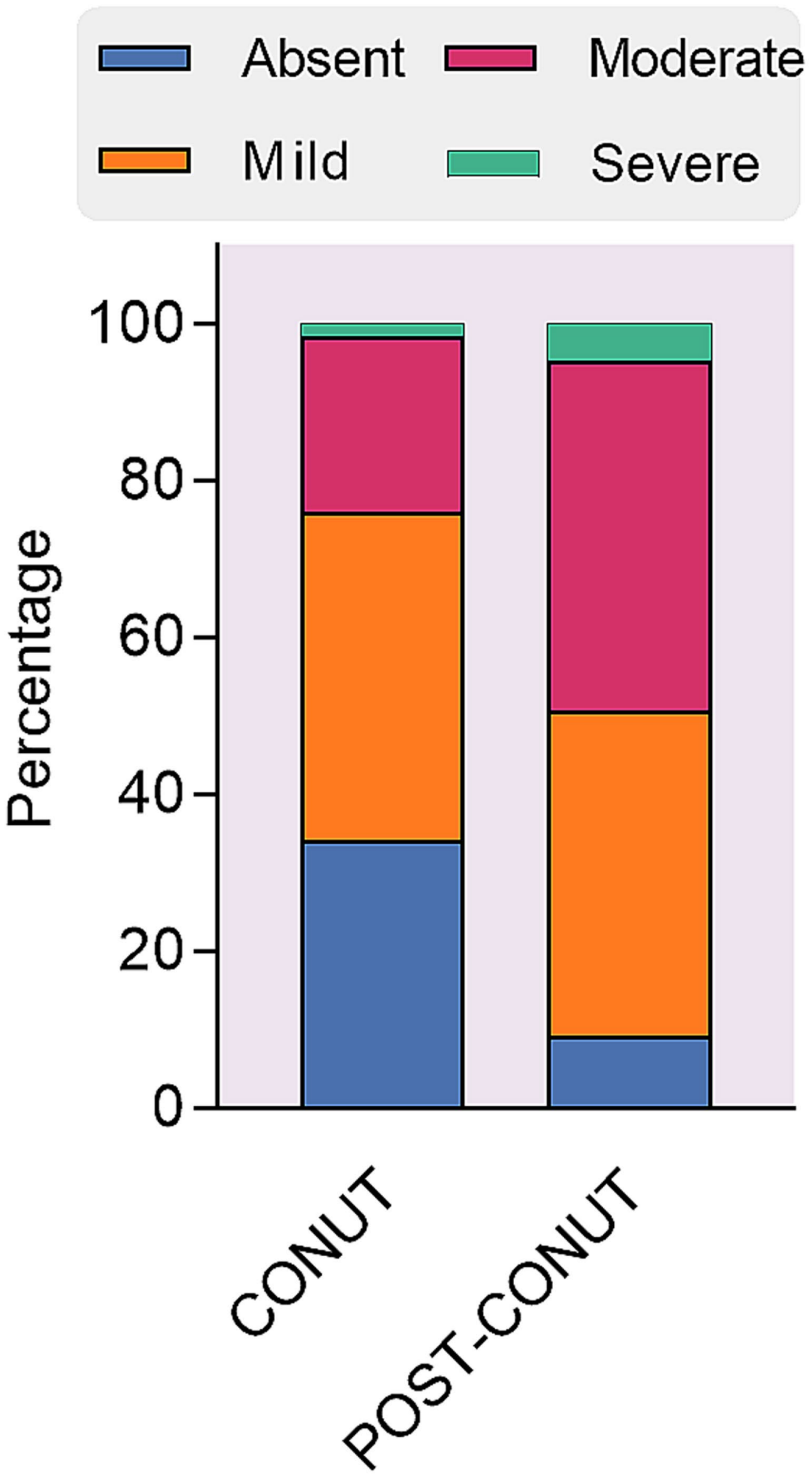
Preoperative and postoperative malnutrition severity percentage bar chart. CONUT, controlling nutritional status score.

**Figure 8:**
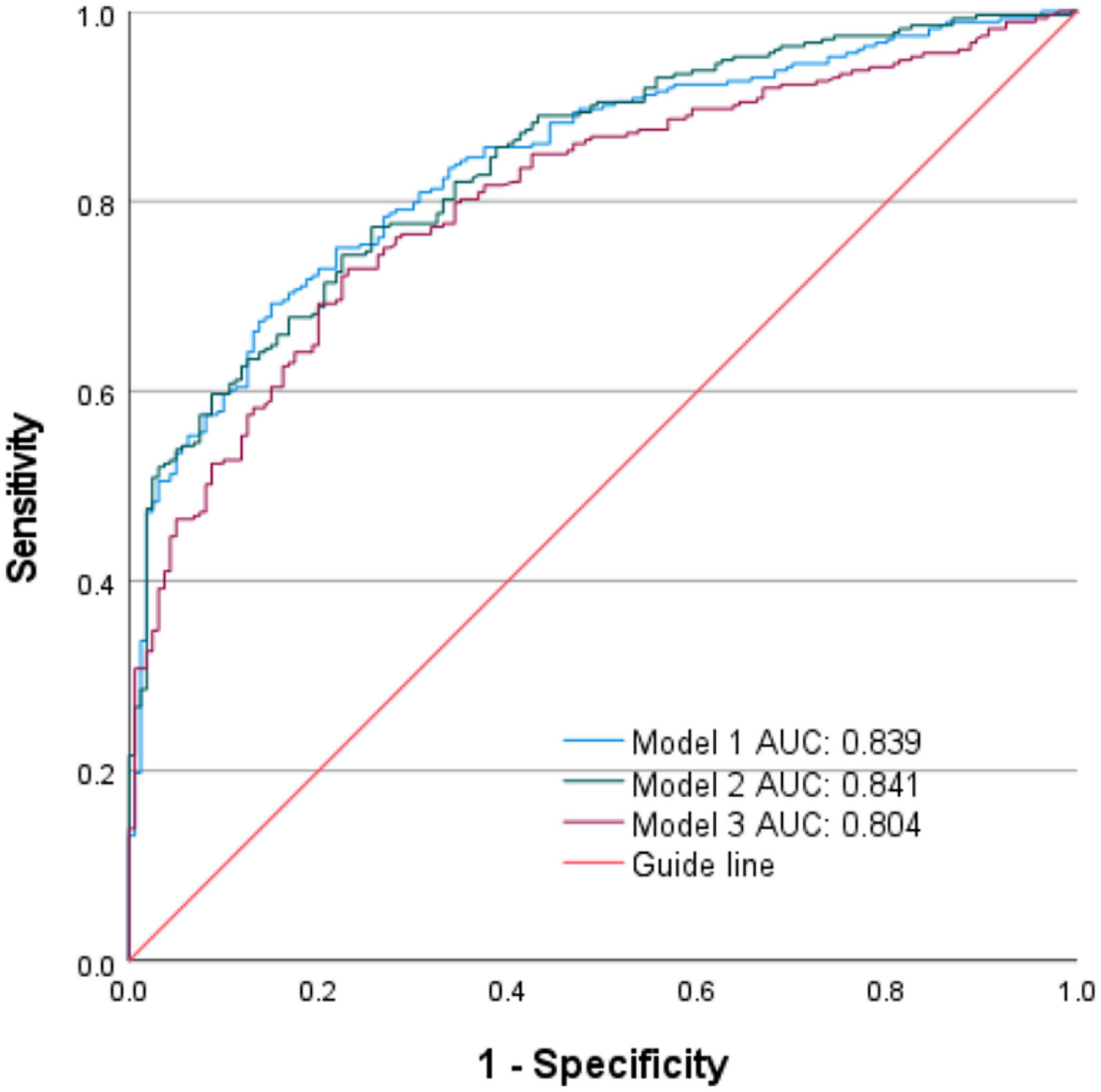
ROC curve of the nomogram model for dynamic changes in CONUT score. Model 1: admission NIHSS + ASPECTS on NCCT + LKW-to-puncture time + preoperative CONUT; Model 2: admission NIHSS + ASPECTS on NCCT + LKW-to-puncture time + postoperative CONUT; Model 3: admission NIHSS + ASPECTS on NCCT + LKW-to-puncture time + ∆CONUT. CONUT, Controlling nutritional status score; NIHSS, National institute of health stroke scale; ASPECTS, Alberta stroke program early computed score; LKW, last known well; NCCT, non-contrast CT.

Postoperative comparison with preoperative levels revealed that the CONUT score increased, lymphocytes decreased, and albumin decreased, all with statistical significance, while total cholesterol showed no statistically significant difference (Figure 6). According to the CONUT score, the preoperative proportions of malnutrition severity were as follows: no malnutrition, 34.2%; mild malnutrition, 41.8%; moderate malnutrition, 22.4%; and severe malnutrition, 1.6%. Postoperatively, the corresponding proportions were: no malnutrition, 9.0%; mild malnutrition, 41.6%; moderate malnutrition, 44.6%; and severe malnutrition, 4.8% (Figure 7). ROC curve analysis indicated that the preoperative nomogram model (model 1) and the postoperative nomogram model (model 2) had a higher AUC than the nomogram model combining ∆CONUT (model 3) (Figure 8). Further DeLong’s test revealed statistically significant differences in AUC between Model 1 and Model 3 and between Model 2 and Model 3, whereas no significant difference was observed between Model 1 and Model 2 (Table 3).

**Table 3:** Comparison of the AUC among model 1, model 2, and model 3.

Model	∆AUC	*Z*-value	95% CI	*P*-value
Model 1—Model 2	−0.002	−0.246	−0.011-0.015	0.806
Model 1—Model 3	0.035	2.951	0.012–0.058	0.003
Model 2—Model 3	0.037	2.922	0.012–0.062	0.003

Model 1: admission NIHSS + ASPECTS on NCCT + LKW-to-puncture time + preoperative CONUT; Model 2: admission NIHSS + ASPECTS on NCCT + LKW-to-puncture time + Postoperative CONUT; Model 3: admission NIHSS + ASPECTS on NCCT + LKW-to-puncture time + ∆CONUT. CONUT, Controlling nutritional status score; NIHSS, National institute of health stroke scale; ASPECTS. Alberta stroke program early computed score; LKW, last known well; NCCT, non-contrast CT; ∆AUC, Difference in the area under the ROC curve.

## 4 Discussion

Our study demonstrated that admission NIHSS, ASPECTS on NCCT, LKW-to-puncture time and CONUT score could serve as reliable predictors of 90-day poor functional outcomes in patients with large vessel occlusion following EVT. The CONUT score-based nomogram model demonstrates comparable predictive performance for predicting adverse outcomes at 90 days in this patient population, whether applied preoperatively or postoperatively. The nomogram model also exhibited excellent discriminative ability in an external validation cohort.

Our study indicates that impaired preoperative nutritional status is common among older patients with LVO undergoing EVT. According to the CONUT score, the proportion of preoperative malnutrition was 65.8%, with moderate to severe malnutrition accounting for 24%. Postoperatively, the prevalence of malnutrition increased significantly to 91%, and moderate to severe malnutrition rose to 49.4%. The CONUT score, which incorporates serum albumin level, lymphocyte count, and total cholesterol level, is a comprehensive indicator reflecting the body’s protein reserves, lipid metabolism, as well as immune and inflammatory status. Previous studies have shown that low serum albumin levels are associated with short and long-term mortality in hospitalized patients (10, 11) and with poor outcomes in AIS patients (12, 13). In ischemic stroke, serum albumin, as a multifunctional protein, exhibits neuroprotective properties such as antioxidant defense, antagonism of leukocyte adhesion, antithrombotic effects, reduced cellular immunity, and sustained neuronal metabolism (14, 15). Lymphocytes are one of the systemic inflammatory markers and play a key role in the pathogenesis of stroke (16). During inflammation, lymphocytes can infiltrate the ischemic area after AIS, leading to the release of pro-inflammatory cytokines and cytotoxic substances, thereby exerting harmful effects (17, 18). Long-term studies have also indicated that lymphocytes are indispensable for tissue repair and remodeling (19, 20). In a Chinese cohort study, a higher lymphocyte count at admission was associated with a reduced risk of death, stroke recurrence, and poor neurological outcome at 1-year follow-up after AIS (21). Total cholesterol is a good indicator of dietary intake. Previous studies have demonstrated that low total cholesterol levels reflect poor nutritional status, increased inflammation, and elevated mortality risk in elderly non-stroke patients (22, 23). For AIS patients, the relationship between low total cholesterol levels and short and long-term mortality has also been reported (24, 25).

Nevertheless, it should be cautiously noted that postoperative changes in CONUT score may not purely reflect alterations in nutritional status. Factors such as acute stroke physiology, anesthesia, surgical stress, systemic inflammation, fluid shifts, and peri-procedural care can substantially influence serum albumin and lymphocyte levels. Brand et al. investigated changes in peripheral immune cell populations, including T lymphocytes, during elective orthopedic surgery under general anesthesia. Their study found alterations in immune cell distribution, with significant changes in T lymphocyte subsets (CD4 + and CD8 + cells) during anesthesia, indicating that anesthesia affects lymphocyte counts (26). Similarly, Liu et al. examined changes in immune cell populations during craniotomy and reported a reduction in immune cells, including lymphocytes, during anesthesia (27). Brand et al. further observed early alterations in circulating lymphocyte subsets and an enhanced pro-inflammatory response, including a significant decrease in lymphocyte counts, during opioid-based anesthesia (28). In addition, the effect of general anesthesia and surgery on serum albumin levels has attracted attention. Research by Hübner et al. (29) suggests that changes in serum albumin may reflect underlying physiological stress or nutritional status related to surgical outcomes. In our cohort, all patients underwent EVT under intubated general anesthesia, and similar changes in lymphocyte count and serum albumin were observed. Therefore, postoperative CONUT score changes represent the combined effects of multiple factors, including nutrition, inflammation, stress, and peri-procedural management. For causal interpretation, these changes should not be simplistically attributed to deterioration in nutritional status. In clinical application, the prognostic value of postoperative CONUT score should be interpreted with consideration of these multifactorial peri-procedural contexts.

Based on the above considerations, we constructed a postoperative nomogram model. ROC analysis showed that the AUC of the postoperative nomogram (0.841) was slightly higher than that of the preoperative model (0.839), but no statistical difference was found between the two AUCs (Table 3). Thus, the CONUT score-based nomogram model demonstrates robust predictive performance both preoperatively and postoperatively; however, its clinical applicability should be interpreted cautiously in the context of multifactorial peri-procedural influences.

Postoperative nutritional decline adversely affects prognosis through multiple mechanisms. From a metabolic perspective, surgical stress triggers a series of physiological responses that alter normal metabolic processes. The primary metabolic consequences of surgical stress include hypermetabolism and catabolism (30, 31). Inadequate energy intake under these conditions leads to delayed nerve repair and increases the risk of complications (32). Postoperative immunosuppression further worsens patient outcomes. The immune response is profoundly influenced by nutritional status (33); malnutrition impairs immune cell function and reduces the body’s ability to fight infections (34). Postoperative inflammation is another critical factor affecting prognosis. Systemic inflammation can trigger neuroinflammation, leading to immune dysregulation and metabolic disturbances, thereby hindering nerve repair processes (35). The interplay among systemic inflammation, immunosuppression, and nutritional status forms a vicious cycle that impedes neurological recovery and worsens overall prognosis. Therefore, addressing malnutrition is essential to mitigating these interrelated pathways and improving clinical outcomes.

Assessing the nutritional status of patients with large vessel occlusion (LVO) has traditionally been challenging, due to the time-sensitive nature of stroke management and variations in patients’ levels of consciousness. Our findings show that the CONUT score is an independent predictor of poor outcomes after EVT in older LVO patients and serves as an objective marker reflecting both nutritional and inflammatory status. Based on these results, we propose a practical clinical pathway: the CONUT score can be routinely calculated upon hospital admission using readily available laboratory data (serum albumin, total cholesterol, and lymphocyte count). For older LVO patients identified as having a poor prognosis based on a CONUT score cutoff value (≥4), we recommend initiating early, individualized nutritional support. To translate this recommendation into clinical practice, we propose the following tiered approach for post-EVT patients based on the established CONUT (4) scoring system (0–1, normal; 2–4, mild malnutrition risk; 5–12, moderate-to-severe malnutrition risk): (1) For patients with CONUT 0–1, routine dietary monitoring is sufficient without the need for specific nutritional support; (2) For those with CONUT 2–3, nutritional support should be provided, targeting an energy intake of 12–25 kcal/kg/day and a protein intake of 1.2–2.0 g/kg/day (36); (3) For patients with CONUT ≥4, a multidisciplinary team-based individualized nutritional support plan is recommended, with close monitoring of swallowing function and metabolic parameters, and timely adjustment of the nutritional delivery route as clinically indicated. This stratified protocol aligns with the principle that nutritional intervention should be proportionate to the severity of malnutrition, thereby maximizing clinical benefits while minimizing unnecessary interventions and potential complications. Notably, these recommendations are derived from our findings and the existing literature, and future prospective studies are warranted to validate their feasibility and efficacy specifically in the EVT population.

### 4.1 Limitations

Our study has several limitations. First, the single-center design limits the generalizability of the results. Although the model was well validated in an external cohort, future validation across different ethnicities and regions is warranted. Second, we only assessed preoperative and postoperative CONUT scores. Given that serum albumin and lymphocyte counts can be influenced by infection or inflammation during hospitalization, dynamic follow-up of these indicators was not performed. Third, several clinically relevant determinants of EVT outcomes were not available for inclusion, including collateral circulation status, dysphagia, post-stroke infections, and post-EVT rehabilitation or nutritional interventions. The absence of these variables is particularly consequential given their complex interplay with nutritional status and prognosis. Specifically, dysphagia not only compromises nutritional intake but also increases the risk of aspiration pneumonia, which may exacerbate systemic inflammation and impede neurological recovery. Post-stroke infections can induce a hypermetabolic state that accelerates nutritional depletion, while the implementation of rehabilitation and nutritional support may directly modify the association between baseline nutritional status and functional outcomes. Furthermore, although collateral circulation status is not directly linked to nutritional pathways, it is a well-established independent predictor of neurological recovery after EVT, and its omission may reduce model specificity. Collectively, the absence of these covariates likely leads to residual confounding through distinct causal pathways. Therefore, our findings should be interpreted as exploratory and hypothesis-generating. To address these limitations, we are designing a prospective, multicenter study with standardized collection of these parameters, which will allow for more robust adjustment and causal inference in future analyses. Fourth, the present study only followed patients up to 90 days after EVT, and therefore long-term prognostic outcomes—such as stroke recurrence, late functional decline, and all-cause mortality—were not assessed. This relatively short follow-up duration limits our ability to evaluate whether the prognostic value of nutritional status persists or attenuates over an extended time horizon. Consequently, our findings are primarily applicable to short-term outcomes and should not be extrapolated to long-term prognosis without caution. To address this limitation, we plan to extend the follow-up of our existing cohort to collect long-term endpoints, including recurrent cerebrovascular events and all-cause mortality, which will enable us to determine the sustained impact of nutritional status on long-term functional independence and survival in future analyses. Finally, Due to the observational study design, our findings demonstrate association rather than causation.

## 5 Conclusion

In this study, we developed a nomogram model based on the CONUT score. This nomogram predicts adverse outcomes at 90 days in this patient population, with comparable predictive performance whether applied preoperatively or postoperatively. External validation confirmed that the model performed well. This visual and individualized prediction model provides clinicians with a simple and intuitive tool that may be of importance for the early detection and identification of patients at risk of poor prognosis.

## Data Availability

The original contributions presented in the study are included in the article/Supplementary material, further inquiries can be directed to the corresponding authors.
