# Supplementary material for: Predictive value of preoperative and postoperative controlling nutritional status scores and their changes in older patients with large vessel occlusion stroke after endovascular thrombectomy
Source: Front Nutr. 2026 Aug 5;13:1838003. doi: 10.3389/fnut.2026.1838003 (PMC13485512; doi:10.3389/fnut.2026.1838003)
Supplement: Supplementary file 1 [file Table_1.DOCX]

**Supplementary table S1.** CONUT score.

| Nutritional Scores | |  | Risk of Malnutrition | |
| --- | --- | --- | --- | --- |
|  | Absent | Mild | Moderate | Severe |
| **CONUT**  ScoreⅠ+ScoreⅡ+ScoreⅢ | 0-1 | 2-4 | 5-8 | 9-12 |
| Albumin, g/L | ≥35 | 30-34.9 | 25-29.9 | <25 |
| **ScoreⅠ** | 0 | 2 | 4 | 6 |
| Total cholesterol, mg/dL | ≥180 | 140-179 | 100-139 | <100 |
| **ScoreⅡ** | 0 | 1 | 2 | 3 |
| Lymphocyte count,×10^9^/L | ≥1.60 | 1.20-1.59 | 0.80-1.19 | <0.80 |
| **ScoreⅢ** | 0 | 1 | 2 | 3 |

**Abbreviations:** CONUT, Controlling Nutritional Status score.
