# Supplementary material for: Predictive value of preoperative and postoperative controlling nutritional status scores and their changes in older patients with large vessel occlusion stroke after endovascular thrombectomy
Source: Front Nutr. 2026 Aug 5;13:1838003. doi: 10.3389/fnut.2026.1838003 (PMC13485512; doi:10.3389/fnut.2026.1838003)
Supplement: Supplementary file 2 [file Table_2.DOCX]

**Supplementary table S2.** Demographic and clinical characteristics of patients in the training and validation cohorts.

| **Variables** | **Total (n=428)** | **Training cohort (n=307)** | **Validation cohort**  **(n=121)** | **P-value** |
| --- | --- | --- | --- | --- |
| **Age, years, M (Q₁, Q₃)** | 71.00 (68.00, 74.00) | 71.50 (68.00, 74.00) | 71.00 (68.00, 74.00) | 0.581 |
| **Gender, n (%)** |  |  |  | 0.845 |
| **Female** | 135 (31.54) | 96 (31.27) | 39 (32.23) |  |
| **Male** | 293 (68.46) | 211 (68.73) | 82 (67.77) |  |
| **Hypertension, n (%)** |  |  |  | 0.920 |
| **No** | 122 (28.50) | 88 (28.66) | 34 (28.10) |  |
| **Yes** | 306 (71.50) | 219 (71.34) | 87 (71.90) |  |
| **Diabetes mellitus, n (%)** |  |  |  | 0.071 |
| **No** | 314 (73.36) | 231 (75.24) | 83 (68.60) |  |
| **Yes** | 114 (26.64) | 76 (24.76) | 38 (31.40) |  |
| **Atrial fibrillation, n (%)** |  |  |  | 0.069 |
| **No** | 290 (67.76) | 200 (65.15) | 90 (74.38) |  |
| **Yes** | 138 (32.24) | 107 (34.85) | 31 (25.62) |  |
| **Hyperlipidemia, n (%)** |  |  |  | 0.847 |
| **No** | 347 (81.07) | 248 (80.78) | 99 (81.82) |  |
| **Yes** | 81 (18.93) | 59 (19.22) | 22 (18.18) |  |
| **Previous ischaemic stroke, n (%)** |  |  |  | 0.429 |
| **No** | 329 (76.87) | 239 (77.85) | 90 (74.38) |  |
| **Yes** | 99 (23.13) | 68 (22.15) | 31 (25.62) |  |
| **Smoking, n (%)** |  |  |  | 0.283 |
| **No** | 271 (63.32) | 190 (61.89) | 81 (66.94) |  |
| **Yes** | 157 (36.68) | 117 (38.11) | 40 (33.06) |  |
| **Alcohol abuse, n (%)** |  |  |  | 0.969 |
| **No** | 348 (81.31) | 250 (81.43) | 98 (80.99) |  |
| **Yes** | 80 (18.69) | 57 (18.57) | 23 (19.01) |  |
| **Medication history, n (%)** |  |  |  |  |
| **Pre-statin use** |  |  |  | 0.578 |
| **No** | 341 (79.67) | 242 (78.83) | 99 (81.82) |  |
| **Yes** | 87 (20.33) | 65 (21.17) | 22 (18.18) |  |
| **Pre-antiplatelet use** |  |  |  | 0.783 |
| **No** | 327 (76.40) | 233 (75.90) | 94 (77.69) |  |
| **Yes** | 101 (23.60) | 74 (24.10) | 27 (22.31) |  |
| **Pre-anticoagulant use** |  |  |  | 0.411 |
| **No** | 377 (88.08) | 268 (87.30) | 109 (90.08) |  |
| **Yes** | 51 (11.92) | 39 (12.70) | 12 (9.92) |  |
| **Admission NIHSS, M (Q₁, Q₃)** | 16.00 (13.00, 20.00) | 16.50 (13.00, 21.00) | 17.00 (12.00, 23.00) | 0.126 |
| **ASPECTS on NCCT, M (Q₁, Q₃)** | 10.00 (9.00, 10.00) | 10.00 (9.00, 10.00) | 10.00 (10.00, 10.00) | 0.319 |
| **Intravenous thrombolysis, n (%)** |  |  |  | 1.000 |
| **No** | 253 (59.11) | 182 (59.28) | 71 (58.68) |  |
| **Yes** | 175 (40.89) | 125 (40.72) | 50 (41.32) |  |
| **TOAST classification, n (%)** |  |  |  | 0.104 |
| **Large-artery atherosclerosis** | 225 (52.57) | 160 (52.12) | 65 (53.72) |  |
| **Cardioembolic** | 149 (34.81) | 114 (37.13) | 35 (28.93) |  |
| **Other etiology** | 54 (12.62) | 33 (10.75) | 21 (17.35) |  |
| **Vascular occlusion site, n (%)** |  |  |  | 0.058 |
| **Internal Carotid Artery** | 153 (35.75) | 105 (34.20) | 48 (39.67) |  |
| **Middle Cerebral Artery** | 208 (48.60) | 161 (52.44) | 47 (38.84) |  |
| **Vertebral Artery** | 21 (4.91) | 12 (3.91) | 9 (7.44) |  |
| **Basilar Artery** | 46 (10.74) | 29 (9.45) | 17 (14.05) |  |
| **Number of Thrombectomy Attempts, M (Q₁, Q₃)** | 1.00 (1.00, 2.00) | 1.00 (1.00, 2.00) | 1.00 (1.00, 2.00) | 0.491 |
| **Salvage therapy, n (%)** |  |  |  | 0.753 |
| **No** | 269 (62.85) | 192 (62.54) | 77 (63.64) |  |
| **Yes** | 159 (37.15) | 115 (37.46) | 44 (36.36) |  |
| **mTICI 2b-3 reperfusion, n (%)** |  |  |  | 0.057 |
| **No** | 47 (10.98) | 28 (9.12) | 19 (15.70) |  |
| **Yes** | 381 (89.02) | 279 (90.88) | 102 (84.30) |  |
| **LKW-to-puncture time, min, M (Q₁, Q₃)** | 280.00 (235.25, 327.00) | 281.00 (227.00, 329.50) | 279.00 (254.00, 325.25) | 0.417 |
| **Puncture-to-Recanalization time, min, M (Q₁, Q₃)** | 101.00 (68.00, 147.50) | 100.50 (68.00, 144.75) | 101.00 (67.50, 154.00) | 0.970 |
| **CONUT, M (Q₁, Q₃)** | 2.00 (1.00, 4.00) | 2.00 (1.00, 4.00) | 2.00 (1.00, 4.00) | 0.599 |
| **CONUT scoring system, n (%)** |  |  |  | 0.187 |
| **absent** | 147 (34.35) | 100 (32.57) | 47 (38.84) |  |
| **mild** | 179 (41.82) | 134 (43.65) | 45 (37.19) |  |
| **moderate** | 95 (22.20) | 70 (22.80) | 25 (20.66) |  |
| **severe** | 7 (1.63) | 3 (0.98) | 4 (3.31) |  |

**Abbreviations:** CONUT, Controlling Nutritional Status score; NIHSS, National Institute of Health Stroke Scale; ASPECTS. Alberta Stroke Program Early Computed Score; NCCT, non-contrast computed tomography; TOAST, Trial of Org 10172 in Acute Stroke Treatment; mTICI, modified thrombolysis in cerebral infarction; LKW, last known well.
